# Ixmyelocel-T, an expanded multicellular therapy, contains a unique population of M2-like macrophages

**DOI:** 10.1186/scrt345

**Published:** 2013-11-01

**Authors:** Kelly J Ledford, Frank Zeigler, Ronnda L Bartel

**Affiliations:** 1 Aastrom Biosciences, Domino’s Farms, Lobby K, 24 Frank Lloyd Wright Drive, Ann Arbor, MI 48105, USA

## Abstract

**Introduction:**

M2 macrophages promote tissue repair and regeneration through various mechanisms including immunomodulation and scavenging of tissue debris. Delivering increased numbers of these cells to ischemic tissues may limit tissue injury and promote repair. Ixmyelocel-T is an expanded, autologous multicellular therapy cultured from bone-marrow mononuclear cells (BMMNCs). The purpose of this study was to characterize further a unique expanded population of M2-like macrophages, generated in ixmyelocel-T therapy.

**Methods:**

Approximately 50 ml of whole bone marrow was obtained from healthy donors and shipped overnight. BMMNCs were produced by using density-gradient separation and cultured for approximately 12 days to generate ixmyelocel-T. CD14^+^ cells were isolated from ixmyelocel-T with positive selection for analysis. Cell-surface phenotype was examined with flow cytometry and immunofluorescence, and expression of cytokines and chemokines was analyzed with enzyme-linked immunosorbent assay (ELISA). Quantitative real-time PCR was used to analyze expression of genes in BMMNCs, ixmyelocel-T, the CD14^+^ population from ixmyelocel-T, and M1 and M2 macrophages. Ixmyelocel-T was cultured with apoptotic BMMNCs, and then visualized under fluorescence microscopy to assess efferocytosis.

**Results:**

Macrophages in ixmyelocel-T therapy expressed surface markers of M2 macrophages, CD206, and CD163. These cells were also found to express several M2 markers, and few to no M1 markers. After stimulation with lipopolysaccharide (LPS), they showed minimal secretion of the proinflammatory cytokines interleukin-12 (IL-12) and tumor necrosis factor alpha (TNF-α) compared with M1 and M2 macrophages. Ixmyelocel-T macrophages efficiently ingested apoptotic BMMNCs.

**Conclusions:**

Ixmyelocel-T therapy contains a unique population of M2-like macrophages that are characterized by expression of M2 markers, decreased secretion of proinflammatory cytokines after inflammatory stimuli, and efficient removal of apoptotic cells. This subpopulation of cells may have a potential role in tissue repair and regeneration.

## Introduction

Macrophages are a diverse population of cells that adapt and respond to a variety of signals, including cytokines and microbial products [1]. Macrophages can be classified based on their functional phenotypes; M1 macrophages are classically activated by proinflammatory cytokines such as gamma interferon (IFN-ɣ) and are T-helper 1 (Th1) associated, whereas M2 macrophages are alternatively activated by cytokines such as IL-4 and IL-13, and are T-helper 2 (Th2) associated [2]. Both *in vitro* and *in vivo* studies have demonstrated that M1 macrophages have an inflammatory phenotype that corresponds with the early phases of tissue injury [1], whereas M2 macrophages have an antiinflammatory and tissue-remodeling phenotype corresponding with the late phases of tissue injury [1,3-8]. M2 macrophages help promote clearance of inflammatory cells and the return of tissue homeostasis [7]. Recently, it has become apparent that these macrophage classifications are extremes of a wide spectrum of possible macrophage phenotypes [8-10].

Several diseases are associated with a defect or alteration in macrophage function [5,6,11]. M2 macrophages are characterized as immunosuppressive and reparative, and have been implicated in stable areas of atherosclerotic lesions, myocardial infarction healing, and skeletal muscle repair [12,13]. Several studies have demonstrated that macrophages are differentially activated during cardiac remodeling after myocardial infarction, with M2 macrophages being involved in the reparative phase [11,12,14]. Studies have also shown that atherosclerotic lesions are characterized by the presence of proinflammatory M1 macrophages that fail to switch to an antiinflammatory and reparative phenotype, thus promoting disease progression [5,15-17]. Therefore, increasing the proportion of M2 macrophages in such disease states could be used to limit tissue injury and promote repair.

Ixmyelocel-T is an expanded, autologous multicellular therapy containing a mixture of cell types cultured from BMMNCs [18-20]. Recent clinical trials evaluating ixmyelocel-T therapy in the treatment of dilated cardiomyopathy and severe peripheral artery disease have shown clinical promise [19,21,22]. Ixmyelocel-T contains a mixture of cells; the process used to generate this cell therapy expands both the CD90^+^ mesenchymal stromal cells (MSCs) and CD14^+^ macrophages, while retaining many of the CD45^+^ cells found in the bone marrow, because the process does not use any purification or enrichment steps, other than phenotypic expansion. The MSCs have been previously characterized both *in vitro* and *in vivo *[18,20]. Although early development of ixmyelocel-T was focused on bone-regeneration studies due to the more obvious expansion of the MSCs which were extremely rare in bone marrow, an initial characterization of the CD14+ hematopoietic cells [20], demonstrated the simultaneous expansion of macrophages, which were primarily responsible for the secretion of high levels of IL-10 and IL-1a receptor antagonist, and were therefore consistent with these macrophages being M2-like.

The purpose of this study was to further characterize ixmyelocel-T macrophages, and determine more precisely their phenotype. The population of macrophages generated in ixmyelocel-T, may be beneficial in the treatment of diseases where tissue remodeling and immunomodulation are key components of successful clinical outcomes. Ixmyelocel-T macrophages are characterized as M2-like with minimal secretion of pro-inflammatory cytokines after inflammatory challenge, as well as efficient removal of apoptotic cells (efferocytosis). Additionally, this data provides evidence that ixmyelocel-T contains a unique M2-like population of macrophages when compared to *in vitro* generated M1 and M2 macrophages.

## Methods

### Cell culture

For the generation of ixmyelocel-T commercially available bone marrow aspirates (Lonza, MD, USA) were obtained from healthy donors under informed consent. A small volume (~50 mL) of whole bone marrow was obtained through needle aspiration of the posterior iliac crest, and stored in heparinized tubes during shipment at ambient temperature to a central processing facility. The mononuclear cell fraction was obtained via an automated, closed-system, Ficoll-based density gradient centrifugation separation process. The isolated mononuclear cells were then transferred to a sterile, single-use cell bioreactor cassette [20,23]. This proprietary system controlled temperature, culture medium exchange, and gas exchange during the culture period. After approximately 12 days, the cells were washed and harvested from the cassette by a multistep, automated process, and ready for experimental study. For the generation of M1 and M2 macrophages, human peripheral blood monocytes from healthy donors were purchased (AllCells LLC., Alameda, USA). The cells were polarized into M1 and M2 macrophages using established protocols [24,25]. Briefly, monocytes were differentiated into macrophages by incubation with 100 ng/mL M-CSF (R&D Systems, Minneapolis, MN, USA) in RPMI-1640 (Invitrogen) supplemented with 20% FBS (Invitrogen) for 7 days. The media was then removed and the macrophages were then polarized with 10 pg/mL LPS plus 20 ng/mL interferon gamma (IFN-ɣ) for M1 polarization, or 20 ng/mL IL-4 for M2 polarization (R&D Systems, Minneapolis, MN, USA) in RPMI-1640 with 5% FBS for 18 hours.

### CD14+ cell purification

CD14+ cells were isolated from ixmyelocel-T by positive selection using MACS beads (Miltenyi Biotec, Bergisch Gladbach, Germany) as per the manufacturer’s instructions. After positive selection, the CD14+ ixmyelocel-T macrophages were transferred to 6-well culture plates for subsequent experiments. Re-analyses of the selected cells by flow cytometry indicated a relatively pure preparation (approximately >90%).

### Flow cytometry

For cell surface staining, erythrocytes were lysed with lysing solution for 10 minutes (Becton Dickinson, Sad Jose, CA). F_c_ receptors were blocked with F_c_ receptor blocking agent (Miltenyi Biotech, Auburn, CA, USA) for 15 minutes at 4°C. Cells were then incubated with surface receptor antibodies for 15 minutes at 4°C, and then washed with phosphate buffered saline (PBS). Cell surface staining was analyzed using the Gallios flow cytometer (Beckman Coulter, Brea, CA, USA). Kaluza software (Beckman Coulter) was used to analyze the acquired data. The following antibodies were used for the analyses: anti-CD14 FITC, anti-CD14 APC, anti-CD206 PE, anti-CD16 ECD, anti-HLA-DR ECD and Annexin V FITC (Beckman Coulter), anti-CD163 APC and anti-CD163 PerCP5.5 (BioLegend, San Diego, CA, USA), and anti-MerTK (R&D Systems).

### Immunofluorescence staining

To visualize alternatively activated macrophages in ixmyelocel-T, cells were seeded in chamber slides and immunostained with CD14, CD90, CD3 antibody (Santa Cruz Biotechnology Inc) for 1 hour at room temperature prior to incubation with fluorochrome-tagged secondary antibody for 1 hour at room temperature. Counterstaining was performed with DAPI to visualize nuclei. Fluorescent microscopy was performed using a Nikon Eclipse TE2000-S Microscope (Nikon, Tokyo, Japan) equipped with a Spot Xplorer Leica digital camera (SPOT, Sterling Heights, MI, USA).

### Real-time quantitative PCR

For real-time PCR analysis, CD14+ selected macrophages from ixmyelocel-T were compared to CD14+ monocytes from the starting BMMNC population, M1 Macrophages, or M2 Macrophages. Total RNA was extracted with the RNeasy Mini Kit (Qiagen, Valencia, CA, USA) and 1 μg of RNA was reverse transcribed using a high capacity cDNA reverse transcription kit per the manufacturer’s directions (Applied Biosciences, Carlsbad, CA, USA). Relative levels of target gene expression were measured on the 7500 Real-Time PCR system (Applied Biosystems). FAM-based Taqman Gene Expression Assay Mix (Applied Biosystems) specific for each gene of interest and Taqman Universal Master Mix (Applied Biosystems) were used. Relative quantification PCR analysis was performed using the ABI 7500 Software (Applied Biosystems). The relative amount of cDNA was calculated by normalization to the relative levels of Gapdh.

### Cytokine analysis

Enzyme-linked immunosorbent assay (ELISA) kits were used to determine the concentrations of the following cytokines: interleukin (IL)-10, IL-1ra, tumor necrosis factor alpha (TNFα), and IL-12 p70 (R&D Systems, Minneapolis, MN, USA). For cytokine analysis of ixmyelocel-T vs. BMMNCs, cells were plated in the presence or absence of 0.1 μg/mL lipopolysaccharide (LPS) overnight. After LPS challenge, supernatants were collected and assayed for cytokines. For cytokine analysis of ixmyelocel-T macrophages vs. M1/M2 macrophages, cells were positively selected using CD14 MACS beads (Miltenyi Biotech) to isolate macrophages. After selection, cells were plated in the presence or absence of 0.1 μg/mL lipopolysaccharide (LPS) overnight. After LPS challenge, supernatants were collected and assayed for cytokines.

### Efferocytosis assay

BMMNCs were cryopreserved for use in the efferocytosis experiments. Frozen BMMNCs were thawed, washed, and then labeled with the lipophilic dye PKH26 as recommended by the manufacturer (Sigma, St. Louis, MO, USA), and apoptosis was induced with 2 μM staurosporine (Sigma). The apoptotic PKH26-labeled BMMNCs were added in a 1:1 concentration to ixmyelocel-T derived from the same marrow donor. For fluorescent microscopy, ixmyelocel-T was labeled with PKH67 as recommended by the manufacturer (Sigma). Ixmyelocel-T was incubated with the apoptotic PKH26-labeled BMMNCs for 3 hours and then washed with PBS. The ixmyelocel-T samples mixed with apoptotic cells were then analyzed by fluorescent microscopy to determine if ixmyelocel-T macrophages ingested apoptotic cells.

### Statistical analysis

Paired t-test was performed to compare results. A *p*-value less than 0.05 was considered statistically significant. Data are reported as mean ± SEM.

## Results

### Ixmyelocel-T contains a population of M2-like macrophages

**Figure 1:**
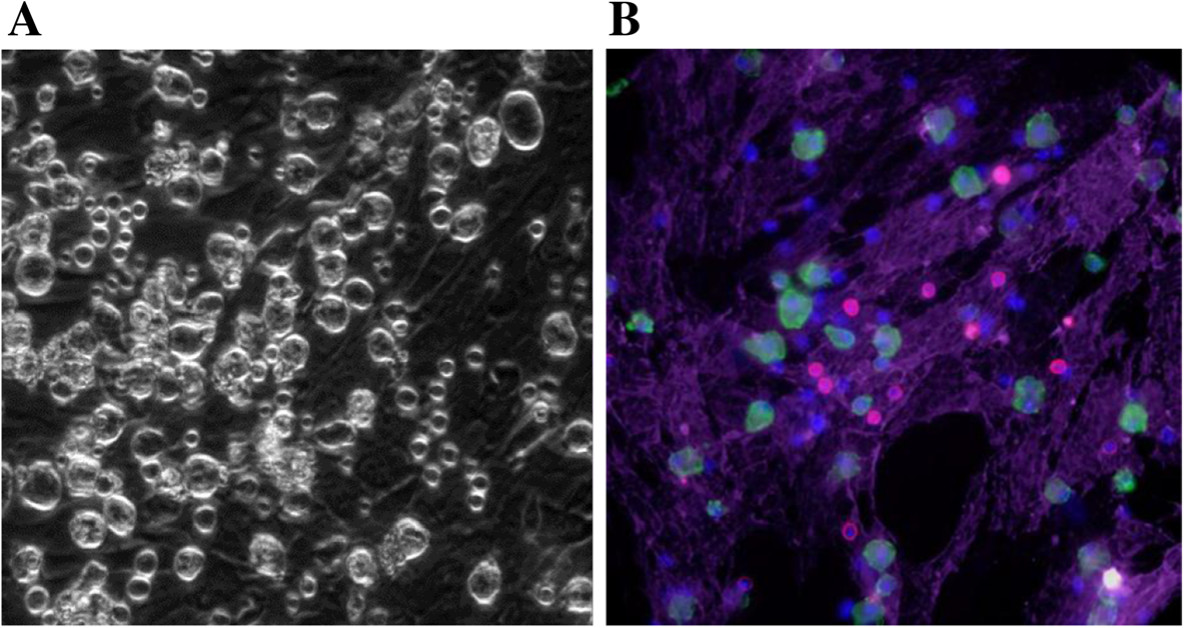
**Ixmyelocel-T contains a mixture of cells, mainly expanded CD14+ macrophages and CD90+ MSC. (A)** Phase imaging depicts the mixture of cells found in ixmyelocel-T. **(B)** Fluorescent imaging of CD90 (purple), CD14 (green), CD3 (red), and nuclei. Magnification: 20 ×.

Ixmyelocel-T is composed of a mixture of cells, specifically this mixture contains myeloid cells (macrophages, granulocytes, monocytes, and mixed myeloid progenitors), lymphoid cells (T cells, B cells, and a mixture of lymphoid precursors) and MSC/stromal cells [20]. As reported previously, the two main cell types expanded from BMMNCs in Aastrom’s manufacturing process are CD90+ stromal cells and CD14+ macrophages [20]. On average there is about a 90 fold increase in CD14+ macrophages in ixmyelocel-T from BMMNCs [20]. Figure 1 displays a microscopy image of ixmyelocel-T, and highlights the two populations which are expanded in this culture process (Figure 1).

**Figure 2:**
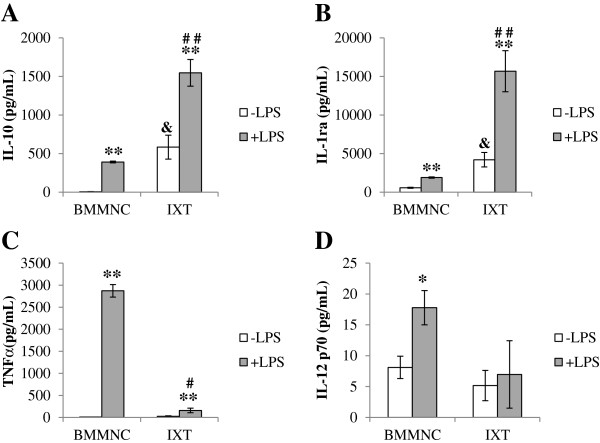
**The expansion process used to produce ixmyelocel-T results in an anti-inflammatory cytokine profile. (A)** IL-10, **(B)** IL-r1a, **(C)** TNFα, **(D)** IL-12 were quantified in BMMNCs and ixmyelocel-T supernatants treated with and without 0.1 μg/mL LPS (n = 5–11). Values are presented as mean ± SEM relative to control, ^*^P <0.01, ^**^P <0.001 vs BMMNC + LPS. ^#^*P* <0.01, ^##^*P* <0.001 vs IXT + LPS. &*P* <0.01 vs BMMNC -LPS. LPS, lipopolysaccharide; SEM = standard error of the mean.

This mixture of cells has previously been presumptively defined as anti-inflammatory, with a majority of the anti-inflammatory cytokines’ secretion being attributed to the expanded population of CD14+ macrophages [20]. The secretory cytokine profile of ixmyelocel-T macrophages has previously been reported to be consistent with a M2-like phenotype since it has been stated that M2 macrophages regulate the inflammatory response by producing anti-inflammatory cytokines including IL-1ra and IL-10 [7,20,26]. This mixed cellular therapy is injected into disease states which are often associated with inflammation; therefore to determine if this anti-inflammatory profile would remain in the face of inflammatory challenge, the cells were stimulated with LPS overnight. These results were compared to BMMNC to determine if this effect is due to the *ex vivo* expansion of the cells. Ixmyelocel-T secreted significantly elevated levels of the anti-inflammatory cytokine IL-10 before (583 ± 154 vs 5 ± 2 pg/ml, *p* < 0.01 compared with BMMNCs, Figure 2A) and after (391 ± 11 vs 1547 ± 173 pg/ml, *p* < 0.001 compared with BMMNCs, Figure 2A) LPS stimulation compared to BMMNCs. Additionally, ixmyelocel-T secreted significantly elevated levels of the anti-inflammatory cytokine IL-1ra before (4207 ± 934 vs 562 ± 68 pg/ml, *p* < 0.01 compared with BMMNCs, Figure 2B) and after (15685 ± 2656 vs 1898 ± 81 pg/ml, *p* < 0.001 compared with BMMNCs, Figure 2B) LPS stimulation compared to BMMNCs. Both ixmyelocel-T and BMMNCs secreted low levels of the pro-inflammatory cytokine TNFα before LPS stimulation (27 ± 11 vs 9 ± 1 pg/ml, *p* = 0.17 compared with BMMNCs, Figure 2C). Ixmyelocel-T secretion of TNFα remained low after LPS stimulation in comparison to BMMNCs (158 ± 52 vs 2872 ± 141 pg/ml, *p* < 0.001 compared with BMMNCs, Figure 2C) compared to BMMNCs. Both ixmyelocel-T and BMMNCs secreted low levels of the pro-inflammatory cytokine IL-12 p70 before LPS stimulation (5 ± 2 vs 8 ± 2 pg/ml, *p* = 0.21 compared with BMMNCs, Figure 2D). Ixmyelocel-T secretion of IL-12 remained low after LPS stimulation in comparison to BMMNCs (7 ± 5 vs 18 ± 3 pg/ml, *p* = 0.07 compared with BMMNCs, Figure 2D) compared to BMMNCs. These results suggest that the expansion process used to generate ixmyelocel-T from BMMNCs generates this anti-inflammatory phenotype.

**Figure 3:**
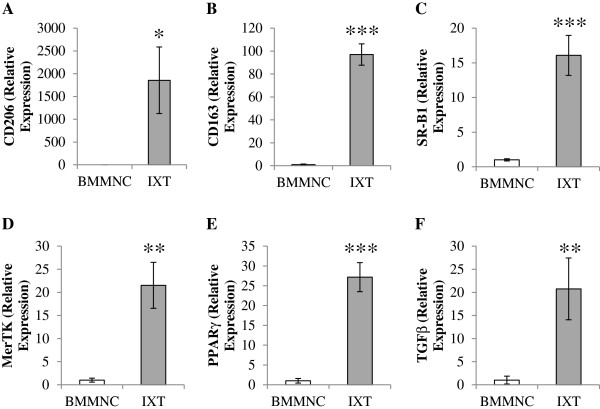
**Ixmyelocel-T macrophages express genes of alternatively activated macrophage phenotype.** Quantitative real-time PCR gene expression analysis of alternatively activated macrophage markers within the ixmyelocel-T macrophages normalized to GAPDH (n ≥ 4). Relative expression of **(A)** CD206, **(B)** CD163, **(C)** SR-B1, **(D)** MerTK, **(E)** PPARγ, and **(F)** TGFβ in CD14+ ixmyelocel-T macrophages. Values are presented as mean ± SEM relative to control, ^*^P <0.05 vs ixmyelocel-T,^**^P < 0.01 vs ixmyelocel-T, ^***^P < 0.001 vs ixmyelocel-T. GAPDH, glyceraldehyde 3-phosphate dehydrogenase; PPAR-γ, peroxisome proliferator-activated receptor gamma; TGFβ, transforming growth factor beta.

Consistent with previously reported findings, quantitative real-time PCR analysis demonstrated that the process used to generate ixmyelocel-T macrophages results in increased expression of M2 macrophage markers. Specifically, ixmyelocel-T macrophages express a statistically significantly higher level of 3 scavenger receptors that are reported to be expressed on M2 macrophages [5,27]: the *mannose receptor (CD206)* (1856 ± 730 vs 1 ± 0.65 relative expression, *p* < 0.05 compared with BMMNCs; Figure 3A), *the haptoglobin-hemoglobin scavenger receptor (CD163)* (97 ± 9.26 vs 1 ± 0.54 relative expression, *p* < 0.001 compared with BMMNCs; Figure 3B), and *scavenger receptor (SR)-B1* (16 ± 2.88 vs 1 ± 0.15 relative expression, *p* < 0.001 compared with BMMNCs; Figure 3C). Ixmyelocel-T macrophages were also found to express significantly upregulated levels of *MerTK* (22 ± 4.98 vs 1 ± 0.43 relative expression, *p* < 0.01 compared with BMMNCs; Figure 3D), a receptor involved in the phagocytosis of apoptotic cells [28,29]. These cells also expressed *PPAR γ* (27 ± 3.68 vs. 1 ± 0.58 relative expression, *p* < 0.001 compared with BMMNCs; Figure 3E), a ligand-activated nuclear receptor which is induced upon differentiation of monocytes into M2 macrophages [1,30]. Expression of *transforming growth factor (TGF)-β*, an indicator of the anti-inflammatory phenotype, was also increased in ixmyelocel-T macrophages (21 ± 6.69 vs 1 ± 0.85 relative expression, *p* < 0.01 compared with BMMNCs; Figure 3F) [31,32]. Together these findings suggest that *ex vivo* expansion produces a CD14+ population of cells with an M2-like phenotype.

**Figure 4:**
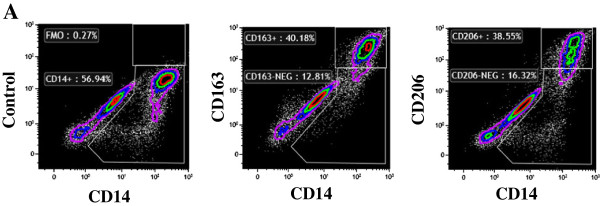
**Ixmyelocel-T macrophages express M2 macrophage surface receptors. (A)** Ixmyelocel-T stained with the M2 macrophage markers CD163 and CD206.

**Figure 5:**
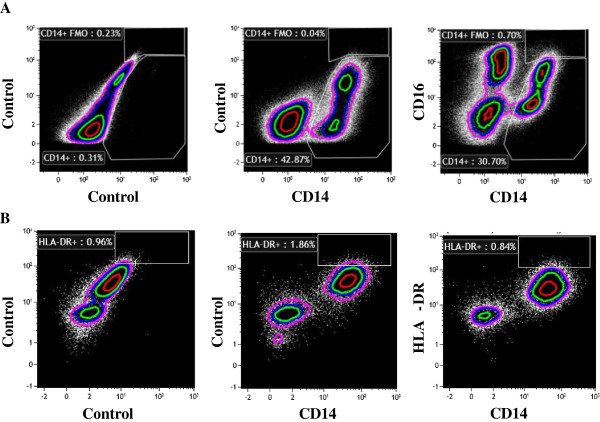
**Ixmyelocel-T macrophages do not express pro-inflammatory macrophage surface receptors.** Flow cytometry of ixmyelocel-T stained with the markers **(A)** CD16 and **(B)** HLA-DR.

Flow cytometry analysis (Figure 4A) confirmed that ixmyelocel-T macrophages express 2 well-known surface receptors of M2 macrophages: CD206 and CD163 [33]. On average 17% of the cells in ixmyelocel-T express CD206 (17 ± 0.6%) and 15% of the cells in ixmyelocel-T express CD163 (15 ± 1.3%). Since ixmyelocel-T consists of a mixture of cells two markers of pro-inflammatory macrophages were also examined. Flow cytometry analysis revealed that the expression of the two pro-inflammatory markers associated with M1 macrophages- CD16 (Figure 5A) and HLA-DR (Figure 5B) is minimal on ixmyelocel-T macrophages. These findings suggest that ixmyelocel-T consists mainly of macrophages with a M2-like phenotype.

### Ixmyelocel-T macrophages are a unique M2-like macrophage

**Figure 6:**
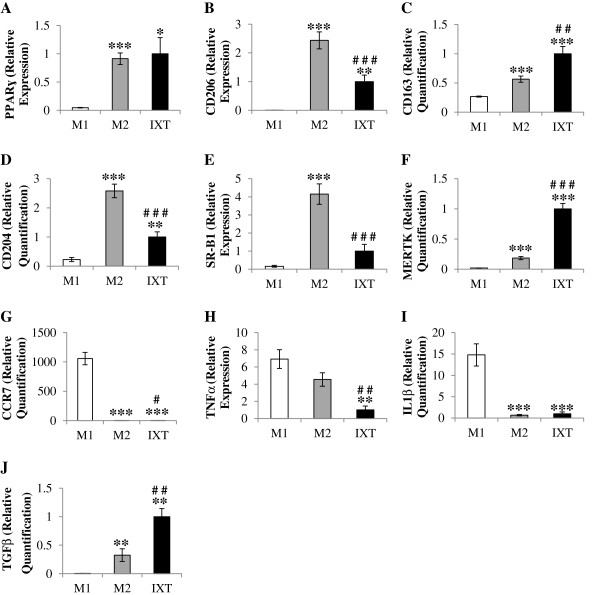
**Ixmyelocel-T macrophages express M2 markers.** Quantitative real-time PCR gene expression analysis of M1 and M2 macrophage markers within the ixmyelocel-T, M1, and M2 macrophages normalized to GAPDH (n ≥ 4). Relative expression of **(A)** PPARγ, **(B)** CD206, **(C)** CD163, **(D)** CD204, **(E)** SR-B1, **(F)** MERTK, **(G)** CCR7, **(H)** TNFα, **(I)** IL-1β, and **(J)** TGFβ in M1, M2, and IXT macrophages. Values are presented as mean ± SEM relative to IXT, ^*^P <0.05,^**^P < 0.01, ^***^P < 0.001 vs M1, ^#^*P* <0.05*,*^##^*P* < 0.01, ^###^*P* < 0.001 vs M2. PPAR-γ, peroxisome proliferator-activated receptor gamma; TGFβ, transforming growth factor beta.

Since ixmyelocel-T macrophages were found to primarily express surface expression of M2 markers, ixmyelocel-T macrophages were further characterized by direct comparison to M1 and M2 macrophages generated *in vitro*. Quantitative real-time PCR analysis demonstrated that ixmyelocel-T macrophages express several M2 Macrophage markers. Ixmyelocel-T and M2 macrophages expressed similar levels of *PPARɣ-* (1.0 ± 0.3 vs 1.1 ± 0.1 relative expression, *p* = 0.42 compared with M2; Figure 6A). M2 macrophages expressed significantly higher levels of *CD206* (1.0 ± 0.2 vs 2.4 ± 0.3 relative expression, *p* < 0.001 compared with M2; Figure 6B), whereas ixmyelocel-T macrophages expressed significantly higher levels of *CD163* (1.0 ± 0.1 vs 0.6 ± 0.1 relative expression, *p* < 0.01 compared with M2; Figure 6C). Ixmyelocel-T macrophages expressed lower levels of the scavenger receptors *CD204* (1.0 ± 0.2 vs 2.6 ± 0.2 relative expression, *p* < 0.001 compared with M2; Figure 6D) and *SR-B1* (1.0 ± 0.4 vs 4.2 ± 0.6 relative expression, *p* < 0.001 compared with M2; Figure 6E) in comparison to M2 macrophages, whereas ixmyelocel-T macrophages expressed significantly elevated levels of the scavenger receptor *MerTK* (1.0 ± 0.1 vs 0.2 ± 0.03 relative expression, *p* < 0.001 compared with M2; Figure 6F). M1 macrophages expressed relatively low expression of all these markers, consistent with previous reports [6,24,34]. Ixmyelocel-T macrophages expressed significantly lower expression of the M1 macrophage markers *CCR7* (1.0 ± 0.4 vs 1058 ± 105 relative expression, *p* < 0.001 compared with M1; Figure 6G), *TNFα* (1.0 ± 0.4 vs 6.9 ± 1.1 relative expression, *p* < 0.01 compared with M1; Figure 6H), and *IL-1β* (1.0 ± 0.4 vs 14.8 ± 2.6 relative expression, *p* < 0.001 compared with M1; Figure 6I). Additionally, ixmyelocel-T macrophages were found to express elevated levels of the reparative cytokine *TGF-β* (Figure 6J). These findings suggest that ixmyelocel-T macrophages are mainly M2-like, as they share little gene expression in common with M1 macrophages. These finding also suggest that ixmyelocel-T macrophages have a unique M2-like profile since they express lower levels of the scavenger receptors *CD204* and *SR-B1*, and elevated expression of *CD163* and *MerTK*.

**Figure 7:**
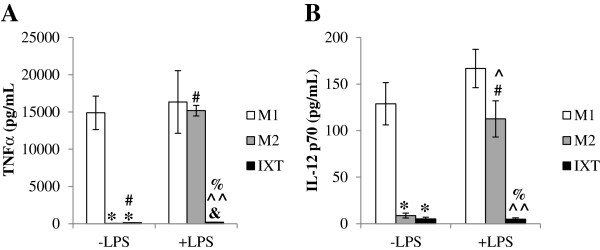
**Ixmyelocel-T macrophages remain anti-inflammatory after inflammatory challenge. (A)** TNFα and **(B)** IL-12 were quantified in M1, M2, and ixmyelocel-T supernatants treated with and without 0.1 μg/mL LPS (n = 4–7). Values are presented as mean ± SEM relative to control, ^*^P <0.001 vs M1. ^#^*P* <0.001 vs M2. &*P* <0.05 vs IXT. ^^^*P* <0.05, ^^^^*P* <0.001 vs M1 + LPS. %*P* <0.001 vs M2 + LPS. LPS, lipopolysaccharide; SEM = standard error of the mean.

M1 and M2 macrophage profiles are described as plastic, with M2 macrophages reported to secrete elevated amounts of the pro-inflammatory cytokines TNFα and IL-12 after stimulation with LPS [26,33]. Since ixmyelocel-T was found to secrete low levels of the pro-inflammatory cytokines TNFα and IL-12 after inflammatory challenge, we challenged M1, M2 and ixmyelocel-T macrophages with LPS overnight to determine the plasticity of their phenotype. Basally, both M2 (29 ± 11 pg/mL; Figure 7A) and ixmyelocel-T macrophages (116 ± 15 pg/mL; Figure 7A) secrete minimal amounts of TNFα, whereas M1 macrophages (14893 ± 2247 pg/mL; Figure 7A) secrete elevated levels of TNFα. Ixmyelocel-T macrophages secrete significantly higher levels of TNFα compared to M2 macrophages (116 ± 15 vs 29 ± 11 pg/mL, *p* < 0.001 compared with M2; Figure 7A). Ixmyelocel-T macrophages secrete lower levels of TNFα compared to M1 macrophages (116 ± 15 vs 14893 ± 2247 pg/mL, *p* < 0.001 compared with M1; Figure 7A). As reported previously [35], after stimulation with LPS M2 macrophages secrete significantly elevated levels of TNFα (15182 ± 718 vs 29 ± 11 pg/mL, *p* < 0.001 compared with M2 -LPS; Figure 7A). Ixmyelocel-T macrophages also secrete significantly elevated levels of TNFα after LPS stimulation (184 ± 27 vs 116 ± 15 pg/mL, *p* < 0.05 compared with IXT -LPS; Figure 7A). However this increase in secretion after LPS stimulation is significantly lower in comparison to M1 (184 ± 27 vs 16342 ± 4200 pg/mL, *p* < 0.001 compared with M1 + LPS; Figure 7A) and M2 (184 ± 27 vs 15182 ± 718 pg/mL, *p* < 0.001 compared with M2 + LPS; Figure 7A) macrophages. Basally, both M2 (9 ± 3 pg/mL; Figure 7B) and ixmyelocel-T (5 ± 2 pg/mL; Figure 7B) macrophages secrete minimal amounts of IL-12 p70, whereas M1 macrophages (129 ± 23 pg/mL; Figure 7B) secrete elevated levels of IL-12 p70. As reported previously, after stimulation with LPS M2 macrophages secrete significantly elevated levels of IL-12 p70 (113 ± 19 vs 9 ± 3 pg/mL, *p* < 0.001 compared with M2 -LPS; Figure 7B) [35]. Whereas, ixmyelocel-T macrophages secrete significantly lower amounts of IL-12 p70 after LPS stimulation compared to both M1 (5 ± 2 vs 167 ± 21 pg/mL, *p* < 0.001 compared with M1 + LPS; Figure 7B) and M2 (5 ± 2 vs 113 ± 19 pg/mL, *p* < 0.001 compared with M2 + LPS; Figure 7B) macrophages. These data suggest that ixmyelocel-T macrophages are not readily polarized to a pro-inflammatory phenotype.

### Ixmyelocel-T macrophages express MerTK, a receptor involved in efferocytosis

**Figure 8:**
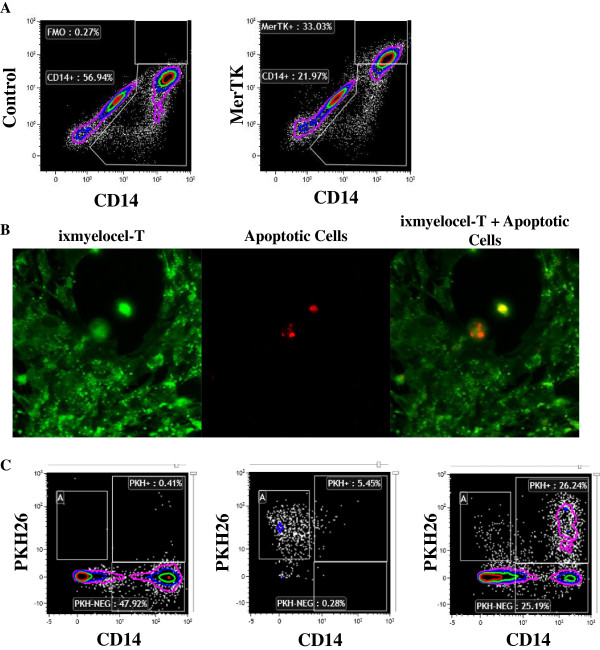
**Ixmyelocel-T alternatively activated macrophages readily phagocytize apoptotic cells. (A)** Ixmyelocel-T stained with MerTK. **(B-C)** Adherent, healthy ixmyelocel-T stained with PKH67 was incubated with PKH26 labeled apoptotic cells. Apoptotic cells were washed away, and healthy ixmyelocel macrophages were analyzed using fluorescence microscopy and flow cytometry. On average 20% percent of ixmyelocel-T CD14+ cells were positive for PKH26+ apoptotic cells after a three hour co-culture apoptotic cells (20 ± 2.9%). (n ≥ 5). Magnification: 60 ×.

M2 macrophages promote the resolution of inflammation by scavenging apoptotic cells, an action termed efferocytosis. Flow cytometry analysis confirmed that ixmyelocel-T macrophages express the surface receptor MerTK (Figure 8A), a receptor involved in the phagocytosis of apoptotic cells [28,29]. To investigate the ability of ixmyelocel-T macrophages to phagocytize apoptotic cells, PKH26-labeled apoptotic BMMNCs were added to ixmyelocel-T in a 1:1 ratio, incubated at 37°C for 3 hours, and then washed with PBS. Fluorescent microscopy and flow cytometry revealed that ixmyelocel-T macrophages ingest apoptotic cells (Figure 8B-C). On average 20% percent of ixmyelocel-T CD14+ cells were positive for PKH26+ apoptotic cells after a three hour co-culture (20 ± 2.9%).

## Discussion

In this report, ixmyelocel-T was found to secrete significantly elevated levels of both IL-10 and IL-1ra before and after LPS stimulation, compared to BMMNCs. Additionally, both ixmyelocel-T and BMMNCs were found to secrete minimal amounts of the pro-inflammatory cytokines TNFα and IL-12. After LPS challenge both ixmyelocel-T and BMMNCs secreted significantly more TNFα, however this increase in secretion was significantly higher in BMMNCs. This expanded population of M2-like macrophages was also found to express significantly elevated levels of the M2 markers *CD206*, *CD163*, *SR-B1*, *MerTK*, *PPARɣ*, and *TGF-β*. Together these findings suggest that the 12 ± 1 day *ex vivo* expansion of autologous marrow generates a population of M2-like macrophages in ixmyelocel-T.

Ixmyelocel-T macrophages were demonstrated to express 2 well-characterized surface markers of M2 macrophages, CD206 and CD163. Whereas surface expression of markers of pro-inflammatory macrophages, CD16 and HLA-DR [36,37], were found to be barely expressed. Studies have demonstrated that macrophages with high expression of CD16 efficiently produce pro-inflammatory cytokines including TNFα and IL-12, while no or very little anti-inflammatory cytokines, whereas macrophages with low expression of CD16 produce low levels of pro-inflammatory cytokines and secrete elevated levels of anti-inflammatory cytokines such as IL-10 [37,38]. These findings suggest that ixmyelocel-T consists mainly of macrophages with anti-inflammatory M2-like phenotypes.

*In vivo*, bone marrow-derived macrophages differentiate from circulating peripheral blood monocytes after migration into tissues, often in response to injury or insult. These macrophages can then be polarized into M1 or M2 macrophages by their microenvironment [39]. As a cellular therapy, ixmyelocel-T macrophages are generated *ex vivo* from bone marrow and directly injected into areas where repair is needed [20,22]. In order to further explore the differences between these cell phenotypes ixmyelocel-T macrophages were compared to M2 and M1 macrophages. Both Ixmyelocel-T and M2 macrophages were found to express similar levels of *PPARɣ*. Ixmyelocel-T macrophages were found to express significantly lower levels of the scavenger receptors *CD206*, *CD204*, and *SR-B1* compared to M2 macrophages. However, ixmyelocel-T macrophages were found to express significantly higher expression of the scavenger receptors *CD163* and *MerTK* compared to M2 macrophages. Ixmyelocel-T macrophages were also found to have significantly higher expression of *TGF-β* compared to M1 and M2 macrophages. When compared to M1 macrophages both M2 and ixmyelocel-T macrophages were found to express significantly lower levels of the M1 markers *CCR7*, *TNFα*, and *IL-1β*. Together these data suggest that ixmyelocel-T macrophages are more M2-like. This data also suggests that ixmyelocel-T macrophages may represent a unique M2-like macrophage, with slightly different expression of M2 markers. Future studies will further examine the unique M2- like phenotype of ixmyelocel-T macrophages to determine just how similar or different they are to M2 macrophages.

Macrophages are plastic cells that can switch from an activated M1 state back to M2, and vice versa depending on specific signals [26,33]. Previous reports have demonstrated that M2 macrophages can be polarized back to a M1 state using pro-inflammatory stimuli such as LPS [35]. To determine if ixmyelocel-T macrophages could also be polarized back to a M1 state in a similar fashion M1, M2, and ixmyelocel-T macrophages were treated with LPS overnight. Before LPS stimulation both M2 and ixmyelocel-T macrophages secreted significantly less TNFα and IL-12 p70 compared to M1 macrophages. However after overnight LPS stimulation, M2 macrophages secreted significantly elevated levels of TNFα and IL-12 p70 similar to M1 levels. This same amount of pro-inflammatory cytokine secretion was not mimicked by ixmyelocel-T macrophages. While ixmyelocel-T macrophages did secrete significantly elevated levels of TNFα after LPS stimulation, this amount of TNFα was significantly lower than that secreted by M2 macrophages. Additionally, ixmyelocel-T macrophages secreted significantly less IL-12 p70 after LPS stimulation compared to M1 and M2 macrophages. These data suggest that ixmyelocel-T macrophages are not readily polarized to a pro-inflammatory phenotype, suggesting that these cells might be beneficial in the treatment of inflammatory diseases. Future studies will further investigate this unique property of ixmyelocel-T macrophages to determine if they can affect the inflammatory state *in vivo*.

M2 macrophages are potent phagocytes that favorably bind and ingest early apoptotic cells [40]. Apoptotic cells need to be removed quickly to prevent the release of tissue-damaging intracellular components that can induce inflammatory responses [28,41]. Efferocytosis itself also triggers pro-resolving signals that promote dampening of the immune response and restoring tissue homeostasis [14]. Defective clearance of apoptotic cells, or efferocytosis, is linked to the progression of several disease states including advanced atherosclerotic lesions, ischemic heart disease, and chronic wounds [28]. Ixmyelocel-T macrophages were found to readily ingest apoptotic cells and express genes for the scavenger receptors that promote the phagocytosis of apoptotic cells. Of specific interest, ixmyelocel-T expresses the scavenger receptor MerTK, which has been implicated in mediating the anti-inflammatory clearance of apoptotic cells [29,42]. The expression of scavenger receptors and their M2-like phenotype predisposes ixmyelocel-T macrophages to be potent phagocytes. This important function of ixmyelocel-T macrophages could potentially promote healing in disease states where efferocytosis is compromised. Specifically, advanced atherosclerotic lesions are characterized by defective efferocytosis, resulting in necrotic core formation [28,42]. In early atherosclerotic lesions, apoptotic cells are rapidly removed by macrophages, which prevents the progression of lesions, but as the disease progresses, apoptotic cells are not removed, resulting in a pro-inflammatory cascade [28]. Efferocytosis is also required in healing the heart after ischemia where removing necrotic debris helps preserve the remaining cardiomyocytes [14]. As such, the macrophages present in ixmyelocel-T may potentially promote tissue repair and regeneration through the removal of necrotic debris.

Several different mechanisms have been reported in the literature that may contribute to the development of M2 macrophages, including ingestion of apoptotic cells, exposure to the Th2 cytokines IL-4 and IL-13, exposure to IL-10, and contact with mesenchymal stem cells [13,27,39,43]. The culture process used to generate ixmyelocel-T results in a population of unique M2-like macrophages. Th2 cytokines are not directly added to ixmyelocel-T during the culture process to produce M2 macrophages [1,31,44]. However, due to the wide mixture of cells found within ixmyelocel-T it is feasible that Th2 cytokines may be generated and secreted by other cells during this 12 ± 1 day process. Ixmyelocel-T is composed of a mixture of cells including macrophages, granulocytes, monocytes, T cells, B cells, and MSC/stromal cells, and cytokine secretion from these different cell types could influence the development of the M2-like phenotype [20]. For example, it has been previously reported that macrophages engage in bidirectional interactions with MSCs, resulting in MSC growth and M2 phenotype polarization [7,12,45]. MSCs have been reported to induce an IL-10 high and IL-12 low M2 phenotype in macrophages [39]. MSCs are expanded during the ixmyelocel-T culture process; the interaction of these cells with macrophages in culture could contribute to the M2-like population of cells found within ixmyelocel-T. Future studies will further examine the relationship between these different cell types to determine if they play a role in the development of this M2-like population of macrophages.

Ixmyelocel-T is currently being evaluated in a Phase 2b clinical trial of advanced heart failure. Tissue recovery after injury is a complex process involving an interplay between macrophages, stem cells, and stromal cells to prevent tissue fibrosis, which can lead to ineffective tissue function [7]. Recent work has highlighted the role of M2 macrophages in these processes [7,17,46]. Macrophages are commonly found in association with areas of fibrosis in cardiac tissue in end-stage heart disease [9], and recent studies have provided evidence that M2-like macrophages have the capacity to remove tissue debris and dampen inflammation in cardiac tissue promoting tissue repair [8]. Additionally, recent studies have found that macrophages found in human carotid artery atherosclerotic plaques are dominated by a M1 phenotype [47], and that the number of pro-inflammatory M1 macrophages found in cardiac adipose tissue correlates with the severity of coronary artery atherosclerosis in human [12]. Therefore, ixmyelocel-T may potentially promote tissue repair and regeneration in these disease states by providing a M2-like population of macrophages described herein. The biological properties of these macrophages in ixmyelocel-T may potentially have clinical utility for tissue repair and regeneration in the disease states where a population of M2 macrophages would be critical.

This study further examined the expanded population of macrophages found in ixmyelocel-T, since macrophages have been reported to play specific and unique roles in tissue regeneration and repair [7,17,46]. Several cellular therapies are currently being explored for the treatment of ischemic cardiovascular diseases where tissue remodeling and immunomodulation are considered key components of successful clinical outcomes. BMMNCs, peripheral blood mononuclear cells, endothelial progenitor cells (EPC), and MSCs are all currently being evaluated in the treatment of ischemic cardiovascular diseases [48]. Tissue regeneration is a complex process involving an interplay between macrophages, stem cells, and stromal cells to prevent tissue fibrosis, which can lead to ineffective tissue function, and it is hypothesized that a mixture of regenerative cells, rather than just a single cell type might be more advantageous [7,20,48,49]. Ixmyelocel-T consists of a mixture of cells generated from BMMNCs, specifically an expanded population of CD90+ stromal cells and CD14+ macrophages which have been characterized with a M2-like phenotype [20]. It is thought that the mixture of cells found in ixmyelocel-T might be more advantageous in long-term tissue regeneration and repair [20,48]. Future studies will further characterize the other cells types that make up ixmyelocel-T in order to highlight the potential roles these other subpopulations might play in tissue repair and regeneration.

There are several limitations to this study. Mainly, the findings and conclusions are based on *in vitro* experiments. The outcome of infiltrating macrophages to areas of inflammatory injury is not fully understood as the inflammatory environment may influence the outcome of the cells. Although we used human cells that would require the use of immune-compromised animals, *in vivo* experiments would strengthen the findings. Additionally, the cells in these experiments were obtained from healthy donors. It would be highly interesting to compare ixmyelocel-T macrophages to the peripheral blood macrophages (polarized M1 and M2) from a diseased patient. Studies have reported that several disease states, such as obesity and type 2 diabetes, affect macrophage phenotype and polarization in some cases limiting M2 polarization capacity [26,50]. Therefore, it would useful to compare ixmyelocel-T macrophages to peripheral blood macrophages in clinically relevant states; especially disease states where ixmyelocel-T is injected as a treatment.

## Conclusion

Our data demonstrate that ixmyelocel-T therapy contains a unique population of M2-like macrophages that are characterized by secretion of anti-inflammatory cytokines and expression of M2 markers- CD206 and CD163. Furthermore, these cells are involved in efficient removal of apoptotic cells and have elevated expression of MerTK, which is imperative in limiting tissue injury and promoting repair. The biological properties of the M2-like macrophages in ixmyelocel-T may have clinical utility for tissue repair and regeneration in the disease states where a population of M2 macrophages would be critical.

## Abbreviations

7-AAD: 7-amino-actinomycin D; BMMNC: Bone marrow blood mononuclear cell; cDNA: Complementary DNA; DAPI: 4′-6-diamidino-2-phenylindole; DMSO: Dimethyl sulfoxide; DNA: Deoxyribonucleic acid; ELISA: Enzyme-linked immunosorbent assay; EPC: Endothelial progenitor cell; FAM: 6-carboxyyfluorescein; GAPDH: Glyceraldehyde 3-phosphate dehydrogenase; IL: Interleukin; LPS: Lipopolysaccharide; MSCs: Mesenchymal stromal cells; PBS: Phosphate buffered saline; PCR: Polymerase chain reaction; PPAR-γ: Peroxisome proliferator-activated receptor gamma; RNA: Ribonucleic acid; SR-A: Scavenger receptor-A; TGF-β: Transforming growth factor; Th1: T helper 1; Th2: T helper 2; TNF-α: Tumor necrosis factor alpha.

## Competing interests

All authors are employees of Aastrom Biosciences, Inc.

## Authors’ contributions

KL conceived and designed research, acquired data (RT-PCR, efferocytosis analysis, ELISA), analyzed and interpreted data, performed statistical analysis, and drafted the manuscript. FZ provided conceptual advice, analyzed data, and participated in the discussion of results. RB contributed to the scientific direction, experimental approach, and interpretation of results. All authors read and approved the final manuscript.
